# Endoscopic treatment of esthesioneuroblastoma

**DOI:** 10.1590/S1808-86942011000200006

**Published:** 2015-10-19

**Authors:** Eduardo Machado Rossi Monteiro, Marcelo Guerra Lopes, Emerson Rodrigo Santos, Caroline Valverde Diniz, Aurélia Silva e Albuquerque, Ana Paula de Aquino Ferreira Monteiro, Mauro Becker Martins Vieira

**Affiliations:** 1 Médico, Residente em Otorrinolaringologia do Hospital Felício Rocho; 2 Médico Especialista, Otorrinolaringologista; 3 Médico Especialista, Otorrinolaringologista; 4 Médico, Residente em Otorrinolaringologia do Hospital Felício Rocho; 5 Médico, Residente em Otorrinolaringologia do Hospital Felício Rocho; 6 Médico Pediatra, Residente em Otorrinolaringologia do Hospital Felício Rocho; 7 Cirurgião de Cabeça e Pescoço Otorrinolaringologista, Coordenador de Clínica de Otorrinolaringologia do Hospital Felício Rocho. Clínica de Otorrinolaringologia e Cirurgia de Cabeça e Pescoço Hospital Felício Rocho

**Keywords:** endoscopy, esthesioneuroblastoma olfactory, video-assisted surgery, paranasal sinus neoplasms, othorhinolaryngologic neoplasms

## Abstract

Esthesioneuroblastoma is an uncommon malignant tumor of the nasal vault. Treatment consists of craniofacial resection. As endoscopic techniques have advanced, this approach has been recommended to avoid morbidity and to reduce costs.

**Aim:**

To evaluate outcomes in patients with esthesioneuroblastoma treated by an endoscopic technique.

**Methods:**

A prospective study of patients diagnosed with esthesioneuroblatoma and treated by an endoscopic technique. The literature over the past 20 years was reviewed for an update on the pathology.

**Results:**

We present 4 patients, 3 males and 1 female, staged according to Kadish and Dulguerov. All were treated surgically with endoscopic techniques, followed by radiotherapy. One patient was also submitted to neck dissection and chemotherapy because of regional metastasis. There were no significant postoperative complications. The mean hospital stay was 3 days; one patient stayed in the ICU for 24 hours after surgery. Follow-up is recent; so far there are no recurrences.

**Conclusion:**

Esthesioneuroblastoma is a potentially curable malignancy. Endoscopic techniques help reduce hospital costs and decrease the morbidity. Adequate margins of healthy tissue are obtained with endoscopic resection, as with craniofacial resection. The literature suggests that outcomes after endoscopic resection are similar to those of the conventional external approach.

## INTRODUCTION

Esthesioneuroblastoma is an uncommon malignancy of the nasal cavity, probably derived from epithelium. This disease generally occurs between the 5^th^ and 6^th^ decades of life; a bimodal distribution (2^nd^ and 6^th^ decades) has been suggested.^1^ Its somber histology has led it to be given several names; at present, two terms are well-established in the literature: esthesioneuroblastoma and olfactory neuroblastoma.^2^, ^3^ It is thought to originate from the neuro-olfactory epithelium in the superior portion of the nasal cavity, the cribriform plate, and the upper-medial surface of the superior turbinate.^1^ This site generally means that symptoms may be nonspecific, resulting in diagnoses made at more advanced phases of the disease (involvement of facial sinuses and the anterior cranial fossa).^4^

This tumor comprises about 6% of cases of paranasal sinus and nasal cavity cancers, and 0.3% of upper aerodigestive tract cancers. About 1,000 cases have been published to date since its first description by Berger et al.^5^

Management of this tumor is uncertain because of the paucity of cases and advances in diagnostic and treatment methods. Current approaches include en bloc surgery, single or combined endoscopic procedures, radiotherapy, and chemotherapy. Although en bloc surgery is considered the standard treatment, it has relatively high postoperative morbidity (about 35%) and mortality (about 2-5%), which has led to a search for new approaches.^6^ Because of increasing command of endoscopic techniques in the nose, these approaches have been advocated to reduce morbidity and costs.^7^

The purpose of this study was to report the experience of our institution in the treatment of this malignancy with endoscopic techniques.

## METHODS

A historical cross-sectional cohort study was carried out at a tertiary level hospital in Belo Horizonte, Minas Gerais state, Brazil, from December 2008 to the present date, August 2010. The institutional review board approved the study (number 323/10).

Patients diagnosed with esthesioneuroblastoma by histological and immunohistochemical studies of biopsy samples, and candidates for surgery were included in our study. The sample comprised 4 patients (3 male and 1 female) aged from 22 to 46 years (mean 33 years). Computed tomography (CT) and magnetic nuclear imaging (MRI) of the facial sinuses was carried out in all patients for surgical planning. Surgical risk assessment was done and a plain chest radiograph was taken to investigate possible metastases. Tumors were staged according to the Kadish et al.^8^ and Dulguerov^9^ & Calcaterra criteria (Tables 1 and 2).

**Table 1:** Staging system according to Kadish et al. and Morita et al.

TYPE	EXTENT
A	Tumor is limited to the nasal cavity
B	Tumor in the nasal cavity and extending to the paranasal sinuses
C	Tumor extends beyond the nasal cavity and paranasal sinuses, involving the cribriform lamina, the skull base, the orbit, or the intracranial cavity
D	Tumor with neck or distant metastases

**Table 2:** Staging system according to Dulguerov et al.

STAGE	FEATURES
T1	Tumor involving the nasal cavity and/or paranasal sinuses (excluding sphenoid), sparing the most superior ethmoidal cells
T2	Tumor involving the nasal cavity and/or paranasal sinuses (including the sphenoid), with extension to or erosion of the cribriform plate
T3	Tumor extending into the orbit or protruding into the anterior cranial fossa, without dural invasion
T4	Tumor involving the brain
N0	No cervical lymph node metastasis
N1	Any form of cervical lymph node metastasis
M0	No metastasis
M1	Distant metastases present

Patients underwent nasosinusal endoscopic surgery to remove the primary tumor; they were informed of the possibility of open surgery if the endoscopic approach was deemed unsatisfactory. A neurosurgery team was ready to intervene if necessary.

Patients were under general anesthesia with controlled hypotension; the head was elevated to reduce bleeding. Local vasoconstriction was attained with cottonoids imbibed in a 1:5000 adrenalin solution, infiltration with xylocaine + 1:100.000 adrenalin, and a 10-minute waiting period. Rigid 4 mm diameter 25 and 45 degree telescopes were used. At first the intranasal tumor mass was debulked to identify the pedicle, as this neoplasm is expansive, rather than infiltrative. The pedicle was then removed with macroscopic free margins, including the mucosa and bony lamina. The lamina papyracea, cribriform lamina, the lacrimal bone, and the posterior septum were removed if appropriate according to the type of lesion. Cottonoid and electrocautery hemostasis was performed. A bipolar cautery was used preferentially in the base of skull and orbit. A mucoperichondrial septal reconstruction flap for the posterior base, described by Hadad, et al.^10^ was used when there was ample exposure of the anterior base of skull. Anterior unilateral tamponade was used for 24 to 72 hours. Nasal cavities were cleaned postoperatively with saline; crusts were removed in the outpatient unit.

Supplementary postoperative radiotherapy was indicated routinely and started from three to four weeks postoperatively. Adjuvant chemotherapy was used in one special case where there was a higher risk of systemic dissemination of the disease.

Nasosinusal endoscopy and imaging (CT and MRI) were used in the postoperative follow-up. Both were done after careful hygiene of the nasosinusal cavities and treatment of infection, if present. Biopsies from tumor sites and/or suspected areas were taken during endoscopy. A plain chest radiograph was done every six months. Survival was not evaluated because the follow-up period was short for a conclusive report.

## RESULTS

### Epidemiology and Staging

All patients complained of progressive nasal block ipsilateral to the tumor, and intermittent headaches. One patient had lateral displacement of the orbit and diplopia; another patient had bilaterally enlarged neck lymph nodes. Inspection revealed a reddish intranasal tumor that bled when manipulated. Three patients were biopsied at our unit. One patient was referred to us with complete histological results. Biopsies were done in the outpatient and hospital settings. There was moderate to intense nasal bleeding, which was controlled with anterior tamponade for 24 hours.

CT was relevant for surgical planning; it showed the anatomy of the facial bones and eventual bony erosions. MRI was employed to assess the true extent of the tumor and to help differentiate neoplastic tissue from secondary sinus disease. MRI was used also to evaluate intracranial and orbitary involvement, and to establish whether there was infiltration or only displacement of these structures because of tumor growth.

Two patients were stage Kadish B and Dulguerov T1N0; one patient was stage Kadish C and Dulguerov T3N0; one patient was stage Kadish D and Dulguerov T2N1 (Table 3).

**Table 3:** Data on patients, treatment and follow-up.

Patient	Age (years)	Sex	Kadish	Dulguerov	Treatment	Follow-up (months)
IGM	40	M	C	T3N0	RE + RT	17
JPP	29	M	D	T2N1	RE + ECB + RT + QT	17
ASM	46	M	B	T1N0	RE + RT	15
LMTP	22	F	B	T1N0	RE + RT	7

Key: RE= endoscopic resection, RT= radiotherapy, ECB= bilateral neck dissection, QT= chemotherapy Source: own data

### Treatment

The same surgical team - experienced in craniofacial resection and nasosinusal endoscopy - carried out the procedures. Endoscopic resection of the primary tumor with equivalent margins to those of open surgery was possible in all patients. There was no need for the neurosurgical team to intervene in any case; the presence of this team in the operating room was important for safety and to provide guidance in cases where the cranial base was more involved.

After debulking, although nasosinusal tumors could appear large, the infiltrative portion was proportionally smaller and generally limited to the lateral or superior walls of the ethmoid. The infiltrated areas were removed and sent separately to the pathology department; margins were assessed, and were free. Intraoperative frozen sections were not carried out.

The anterior cranial base had to be opened - with exposure of the brain - in one patient. It was reconstructed with temporal fascia and a posterior nasoseptal rotation (described by Hadad et al.).^10^

The patient with neck metastases underwent bilateral neck dissection and temporary tracheostomy, and was the only patient that was sent to the intensive care unit in the immediate postoperative period.

The hospital stay was from 2 to 5 days. All patients were treated with postoperative supplementary external radiotherapy, which was started in the third to fourth week after surgery. The patient with neck metastases underwent chemotherapy with radiotherapy, as the risk of dissemination of the disease was higher in this case.

### Complications after surgery

The postoperative morbidity related to endoscopy was minor. There were no significant complications such as bleeding, meningitis, cerebrospinal fluid leak, or altered vision. Two patients developed non-obstructive intranasal synechiae due to therapy. All patients had intranasal crusts for prolonged periods. The patient that underwent neck dissection presented a cervical node during follow-up; it was removed and diagnosed as an inflammatory granuloma, probably suture-related.

### Follow-up

Postoperative follow-up ranged from 7 to 17 months (mean - 14 months). Until the present time no tumors have recurred. One patient has not made the regular visits because of the distance between the hospital and the patient's household (Amapá state). The remaining three patients have been controlled with postoperative imaging and nasosinusal endoscopy with multiple biopsies; these were negative.

## DISCUSSION

**Figure 1:**
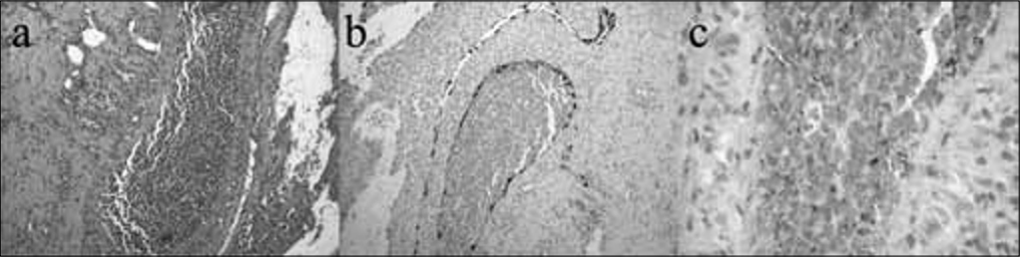
Histology and immunohistochemistry of a surgical specimen (esthesioneuroblastoma). Key: HE-stained histology of a surgical specimen (a) and positive immunohistochemistry for S-100 (b) and synaptophysin (c) markers.

Source: own data.

Esthesioneuroblastoma is an uncommon disease; its symptoms are similar to those of common conditions, such as chronic sinusitis and nasal polyps, which complicates an early diagnosis. The main reported symptoms in our series were nasal block and headache; contrary to other papers,^11^, ^12^, ^13^ epistaxis was infrequent. The differential diagnosis is made with other nasosinusal tumors, such as the inverted papilloma and squamous cell carcinomas.^14^ An early biopsy is recommended in suspect lesions; this procedure is done after tomography of the facial sinuses, which provides guidance for the biopsy site and helps reduce the complication rate. Biopsies should be done in a hospital setting because of the risk of hemorrhage and the need for aggressive tamponade. The histological characterization of esthesioneuroblastoma is difficult; in general, immunohistochemical methods are employed^3^ (Fig. 1).

We applied the Kadish et al. and Dulguerov & Calcaterra staging method in our patients. These criteria are based on preoperative image exams (CT and MRI) to inform the surgeon on the method and prognosis, and to support publishing and research. There is no consensus in the literature about which method is best. A few series appear to suggest that the Dulguerov system is more accurate in predicting survival.^9^ There is a further staging method proposed by Hyams,^15^ which is based on the histology of the disease. Staging yields prognostic information, and ranges from I to IV; grade I refers to patients with good follow-up, and grade IV refers to patients who die because of the disease. Regardless of the staging method, published papers show that the prognosis is worse in proportion to the extent of the tumor, local invasion, and regional or distant metastases, regardless of the treatment.^16^ Our study shows that different staging approaches may be successfully adopted for esthesioneuroblastoma in endoscopy.

**Figure 2:**
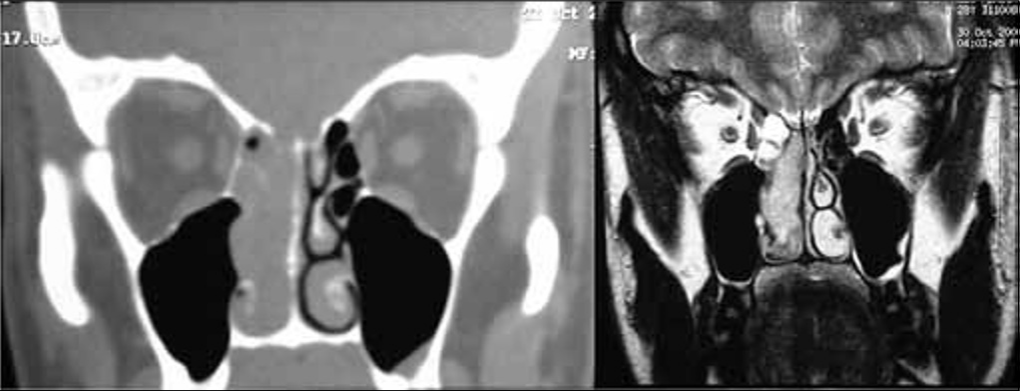
Preoperative CT and MRI of the patient JPP; stage Kadish D and Dulguerov T2n1.

Source: own data.

**Figure 3:**
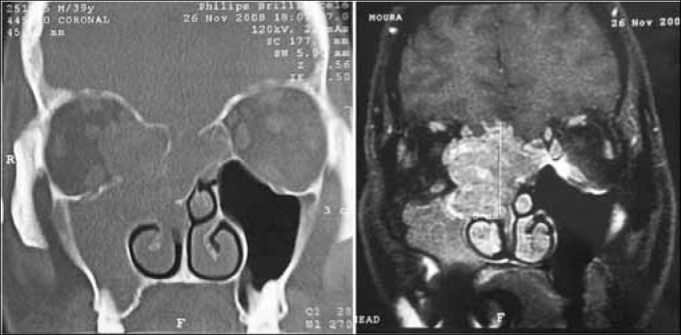
Preoperative CT and MRI of patient IGM, stage Kadish C and Dulguerov T3n0.

Source: own data.

In our experience, MRI was useful to: 1) establish the true extent of the disease and its infiltrative or compressive nature relative to the orbit and the intracranial area; and 2) to differentiate this cancer from secondary sinus disease (Fig. 2). Such information was essential for endoscopic surgery planning, but did not assure that procedures would be successful. The possibility of open surgery should be considered, and the surgical team should be prepared for this option. A multidisciplinary team - including a neurosurgeon - is paramount for advances in endoscopic surgery relative to procedures in the skull base. Although the neurosurgical team was not required in our series, the presence or availability of these professionals increase our sense of safety during surgery and when dealing with postoperative events. One of our patients, staged as Kadish C, presented CT and MRI images suggesting invasion of the orbit and anterior skull base (Fig. 3). During surgery, the orbit was not invaded and the lamina cribosa was involved but without invasion of the dura. There are reports of poor correlation between preoperative CT and MRI and intraoperative findings relative to other nasal tumors, such as in advanced stages of the nasoangiofibroma.^17^ This has not been described or investigated in esthesioneuroblastoma cases.

Significant advances have taken place in nasosinusal endoscopy in the past few years. At first, endoscopy was used for the surgical treatment of infection and inflammatory processes. As proficiency in the techniques increased and appropriate materials were developed, new applications arose, such as endoscopic dacryocystorhinostomy, inferior-medial decompression of the orbit, endoscopic closure of cerebrospinal fluid leaks, among others. The possibilities for endoscopy seem unlimited.

At present, endoscopic techniques have been applied to the treatment of nasosinusal and skull base neoplasms.^7^, ^10^, ^18^, ^19^, ^20^ The indications and limitations of these techniques remain controversial, and need to be further defined, not only what may be done, but especially when.

Stammberger et al.^18^ first published (in the English literature) their experience with endoscopic treatment of nasal malignancies, including esthesioneuroblastomas. Endoscopic resection is indicated for tumors contained within the nostrils and paranasal cavities not deeply infiltrated into the orbit, the pterygopalatine fossa, or the posterior wall of the frontal sinuses.^7^, ^18^ Preoperative CT and MRI are essential for surgical planning.^11^, ^12^ Minor complications such as cerebrospinal fluid leaks and intraorbitary hemorrhage have been described in this technique; the risk is similar to that of endoscopy in chronic sinusitis.^7^

Several authors still defend en bloc craniofacial resection as the treatment of choice for tumors in general.^21^, ^22^ The rationale is that this traditional technique yields safer free margins and consequently fewer recurrences, as well as a more reliable reconstruction of the skull base. This procedure, however, is surgically traumatic; healthy structures are resected to provide access to the surgical field, the duration of surgery is longer, and intensive postoperative care is required. Cerebrospinal fluid leaks, frontal abscesses, pneumocephalus, hydrocephalus, intracranial hemorrhage, subdural hematoma and hygroma, mucoceles, diabetes insipidus, and amaurosis due to thromboembolic disease have been described as complications of open surgery.^23^, ^24^ These complications are potentially avoidable when using endoscopy. In our opinion, an open approach should be employed in selected cases where there is significant local invasion.

Two points need to be demonstrated for endoscopic techniques to be accepted in the treatment of nasosinusal tumors. Firstly, the technique should not compromise the radicality of resection, and recurrences should be similar to the conventional technique. Secondly, it should have significant advantages relative to the conventional technique. We believe this is the case in the treatment of esthesioneuroblastomas. As seen in our series, this tumor is more expansive than infiltrative. After debulking, it was clear that the infiltrated area - and thus the origin of the tumor - is relatively small compared to the total extent of the tumor. En bloc craniofacial resection would have sacrificed healthy tissues in our cases, but would not have increased the safety margins. Meticulous removal of the area of interest - the tumor site - is possible with endoscopic surgery. Other advantages of endoscopic over conventional surgery are: 1) it avoids retraction of frontal lobes, 2) it avoids esthetic and functional loss because of transfacial approaches, 3) it allows difficult areas to be visualized, and 4) it reduces the recovery period and hospital stay of patients. The skull base may be reconstructed endoscopically, if needed.

Radiotherapy was used in all cases, as would have been done if patients had undergone craniofacial resection. The proximity of noble structures - the brain and the orbit - does not allow ample safety margins, which justifies routine adjuvant radiotherapy. Two methods may be used: the external conventional or guided stereotaxic approaches. Compared to the conventional method, stereotaxic radiotherapy was superior in the treatment of esthesioneuroblastoma because of more effective local action and lower morbidity.^25^ At present, it is indicated - as presented in the literature - for patients where surgical resection was incomplete or residual disease is present.^26^ A few authors have recommended this treatment in advanced stages of the disease (Kadish B or D) even after complete resection, for improved local control.^27^ Its use alone is not recommended; published papers have shown that survival is increased when combined therapy is used.^28^ In spite of these advances in surgery and radiotherapy, local and regional recurrence remains a major challenge.^29^

Neck metastases may be found in up to 5% of patients at the diagnosis.^12^ These patients require neck dissection and/or radiotherapy. A review of the literature revealed that up to 23% of patients may have neck lesions as the disease progresses.^30^ Management of negative necks in esthesioneuroblastomas is controversial; some authors have suggested that radiotherapy or elective neck dissection is required, especially if there is significant local invasion.^30^ Furthermore, when clinically apparent, the presence of enlarged neck lymph nodes is associated with distant metastases.^31^ However, as neck disease may take about two years to develop, most authors do not recommend elective treatment of a negative neck.^32^ Regional and distant metastases drastically reduce survival. The high rate of regional metastases described in the literature contradicts the statement that esthesioneuroblastomas are tumors with low malignant potential.^33^ The patient that was staged Kadish D and Dulguerov T2N1 in our sample was treated according to the protocol of renowned international institutions.^10^, ^30^

Our follow-up period is short and the sample is small for survival calculations and the prognosis. At this time, no tumor has recurred, and the success of intraoperative endoscopic resection is encouraging. As in other series, this fact suggests a promising future for this approach.

## CONCLUSION

Esthesioneuroblastoma is a potentially curable malignancy with surgery and radiotherapy. Endoscopic techniques result in significant esthetic and functional gains, reduced recovery times, lower costs, and less morbidity and mortality compared to the conventional approach. It is possible with endoscopy to attain similar margins to those of conventional surgery. Long term results, as described in the literature, are similar to those of the conventional treatment. Our follow-up is short, not allowing any conclusion on the prognosis.
